# ‘I'm not just a number on a sheet, I’m a person’: Domiciliary care, self and getting older

**DOI:** 10.1111/hsc.12921

**Published:** 2019-12-12

**Authors:** Suzanne Hughes, Sarah Burch

**Affiliations:** ^1^ Faculty of Health, Education Medicine and Social Care Anglia Ruskin University Cambridge Cambs UK

**Keywords:** ‘domiciliary care’, ageing, identity, self

## Abstract

Social care funding is reducing in spite of a growing older population. Within this context, domiciliary services are increasingly failing to deliver care that respects the individuality and heterogeneity of older people. To date, there has been limited research in the U.K. that explores, from the older person's perspective, how care practices interact with self.

Using biographical‐narrative methodology, this study takes a constructionist approach to understand the individual's lived experience of care and how it interacts with sense of self. A three‐stage model of data collection was used, beginning with a narrative biographic enquiry, exploring with participants (65 yrs +, *n* = 17) their journeys into care and any possible relationship to personal identity. Stage 2 involved a two‐week period of diary completion, with participants recording daily reflections on their care experiences. In stage 3, a semi‐structured interview explored the diary entries, linking back to the narrative biographic enquiry to reveal ways in which specific care practices interacted with the sense of self.

The findings reveal that a strong relationship between older person and formal carer, forged through familiarity, regularity and consistency, plays a significant role in promoting feelings of autonomy. Furthermore, such relationship mediates against the loss of executional autonomy that often accompanies increasing disability. Maintaining autonomy and control was a recurring theme, including in relation to home, privacy and dignity. Feelings of autonomy are also promoted when formal carers understand the unique ways in which individuals experience ageing and being in the cared‐for relationship.

This paper suggests that a care approach should be based on two tenets. First, a knowledge and insight into the importance of understanding and respecting the older person's continuing development of self, and second applying this knowledge to care through a positive, stable and consistent relationship between the older person and the carer.

**What is known**

- Domiciliary care does not always support older people's autonomy.
- Continuity of formal carers is problematic and provision is often basic and restricted.
- Care services can treat older people as a homogenous group.

**What this paper adds**

- Care is enhanced through understanding the self, different experiences of ageing and the value the older person places on home space.
- Carer consistency supports development of valued relationships.
- Autonomy is promoted when timing of calls is well‐managed and care is flexible.

## 1 INTRODUCTION

This study aims to understand older people's experiences of domiciliary care and whether care practices have implications for their sense of self or personhood. The current emphasis on ageing in place is only supportable if appropriate care is available (Sixsmith & Sixsmith, 2008). However, to date there has been comparatively little research on the social relationships of domiciliary care and older people's sense of identity within these relationships.

### 1.1 Background

Domiciliary care services in the UK are under pressure. Austerity measures have resulted in a real‐term 7% reduction in social care budgets, with gross spending on adult social care services falling from £19.1 billion in 2009/10 to £17.8 billion in 2016/17 (Nuffield Trust, 2017). Yet the older population is growing, with the proportion of people aged over 65 expected to rise across the UK from 18% in 2016 to 24.7% by 2036 (ONS, 2017). The Local Government Association's Well‐being Board (2018) expressed serious concerns that a growing funding crisis will jeopardise local authority services.

It is therefore unsurprising that much research has focused on the commissioning, management and quality of care (Francis & Netten, 2004; Gethin‐Jones, 2012; Glendinning, Clarke, Hare, & Maddison, 2008; Patmore & McNulty, 2005; Qureshi & Henwood, 2000). However, care in the home can also be considered in terms of how it interacts with an older person's sense of self, with a particular emphasis on whether it supports autonomy, as advocated within a person‐centred care philosophy (Care Act, 2014). Collopy (1988) distinguishes between decisional autonomy and executional autonomy, that is the ability to make decisions about one's care and then to carry them out independently. Collopy (1988) suggests that older people see independence and autonomy not as a measure of their functional ability, but rather the extent to which they have control over their world and how seamlessly their care enables this. When an older person requires personal care, they may also begin to lose the opportunity to execute decisions personally (Equality & Human Rights Commission, 2011; Karlsson, Edberg, Jakobsson, & Hallberg, 2013; Sykes & Groom, 2011). Although community‐based care is commonly assumed to promote choice and control, Boyle's (2004) study found that older people receiving domiciliary care perceived themselves as having less decisional autonomy than those in institutional care. Furthermore, Boyle (2004) suggests that restrictions on decisional autonomy can have more impact on mental ill‐health than physical impairment and can contribute to feelings of hopelessness and depression. Healey‐Ogden (2014) argues that caregivers' entry to the home can feel invasive and challenge personal identity. Aronson (2002) states that older people want to take charge of their care, but struggle to do so in the context of service rationing, leading to resignation and limited expectations.

Thus, this study explores, from the older person's perspective, the specific aspects of formal care that promote autonomy and respect the self in the home. There are multiple definitions of the concepts of self and identity in the literature, which take account of experiences such as ageing, context and social relationships in various ways. This study is underpinned by two theoretical approaches from Erikson (1982) and Stryker and Statham (1985). Erikson's life stage theory (1982) proposes that the ego unfolds as an individual moves through the stages of life. Each new stage presents disequilibrium and challenges that the ego must master. How these challenges are tackled shapes how well the individual navigates later stages of life. For example, Hvalvik and Reierson (2011) found that older people receiving care at home were happier when they had reached a point of acceptance. Acceptance was attained through flexibility, recognition and hope, from valuing and understanding their selves, and from how selves were seen in the context of past experiences.

However, concepts of self can also be considered as socially constructed; the transient self reconstructs in response to the social context. Stryker and Statham (1985) argue that the self is shaped through roles that are played out, with each bringing forth a role identity. The ‘social self’ is realised through interaction with others, and identities are developed and maintained within role relationships. For example, placing this in the context of care, Janssen's , Regenmortel, and Abma (2011) qualitative study with older people receiving care in the community reports participants placing great importance on mutual responsibility and solidarity. In spite of their own difficulties, they felt empowered by helping others, and being part of the community was a source of strength and agency.

This study's conceptual framework thus drew upon Erikson's life stage theory for insight into the older person's continuing development of self, and social constructionist role theory to understand how identity is realised through relationships within the context of care. This framework informed exploration of the following research questions:

1. How are domiciliary care practices experienced by older people?
2. How do care practices link to autonomy and how important is this for sense of self?

## 2 METHODS

The research was undertaken as part of a doctoral study. It employed an in‐depth qualitative approach using biographical‐narrative methodology. There were three stages of data collection:

- a biographical‐narrative interview, exploring participants' experiences of formal care and any interaction with sense of self
- a care diary
- a follow‐up interview to explore emerging issues and how care episodes related to individual selves, needs and values

### 2.1 Participants

SH spoke about the study and invited participants at various local organisations, including day centres, user groups and forums. Some third sector organisations also promoted the study in their magazines, inviting interested parties to contact SH directly. Inclusion criteria were as follows:

- Men and women aged 65+
- In receipt of formal care in their own homes (typically that which is paid, regulated and monitored, rather than informal care)
- Living in the Eastern region of England
- Able to communicate in English, both verbally and in writing

Day centre managers acted as gatekeepers. While the study aimed to be inclusive, only those who had the capacity to understand the purpose of the research and to recall events were put forward. Fourteen participants were recruited through day centres, one via a magazine and two through users' forums**. **The sample, as detailed in Table 1, comprised men (*n* = 3) and women (*n* = 14) aged 67 – 92 with a range of disabilities. They all required support with activities of daily living, such as washing, dressing, toileting and taking prescribed medication. Some were severely disabled and required intensive support, while others needed lower level input because they lacked informal care.

**Table 1:** The sample demographic

Name (pseudonym)	Gender	Age	Living alone or with spouse	Health status and Care needs	Recruitment	Biographical‐narrative	Diary	Follow‐up interview
Sarah	Female	72	Alone	Osteoarthritis, leg ulcers. Limited mobility. 3 care visits a day for general and personal needs	Day Centre	✓	✓	✓
Margaret	Female	73	Alone	Osteoarthritis, diabetes, spinal injury. Limited Mobility. 5 care visit a week for general needs	Day Centre	✓	✓	✓
Ellen	Female	92	Alone	Heart condition, diabetes, leg ulcers. Limited mobility. 1 care visit a day for general and personal needs	Day Centre	✓	X	X
Clare	Female	82	Alone	Multiple Sclerosis, stroke. Mobile. 2 care visits a week for general support	Day Centre	✓	X	✓
Pauline	Female	85	Alone	Osteoporosis. Limited mobility. 1 care visit a day for general support and occasional personal needs following falls.	Magazine	✓	✓	✓
Susan	Female	72	Spouse	Spinal injury, Chronic Obstructive Pulmonary Disease Cannot walk unaided. 4 care visits a day for general and personal needs	Forum	✓	✓	✓
Paul	Male	86	Spouse	Stroke, spinal injury. Cannot walk unaided. Confined to bed 24 hrs a day. Requires 4 care visits a day for general and personal needs	Forum	✓	X	X
Karen	Female	67	Alone	Parkinson's disease. Cannot walk unaided. 3 care visits a day for general and personal needs	Day Centre	✓	✓	✓
Janet	Female	82	Alone	Multiple Sclerosis. Cannot walk unaided. 4 care visits a day for general and personal needs	Day Centre	✓	✓	✓
Davina	Female	71	Alone	Stroke. Cannot walk unaided. 3 care visits a day for general and personal needs	Day Centre	✓	X	X
Vera	Female	67	Alone	Stroke, diabetes, leg ulcers. Limited mobility. 3 care visits a day for general and personal needs	Day Centre	✓	X	X
Maud	Female	92	Alone	Osteoporosis and partially blind. Limited mobility. 3 care visits a day for general and personal needs	Day Centre	✓	X	X
Eva	Female	92	Alone	Heart condition. Limited mobility. 1 care visit a day for medication management	Day Centre	✓	X	X
Gillian	Female	90	Alone	Double hip replacement. Limited mobility. 1 care visit a day for general and personal needs	Day Centre	✓	X	X
Maurice	Male	74	Spouse	Osteoarthritis, asthma, epilepsy. Cannot walk unaided. 4 care visits a day for general and personal needs	Day Centre	✓	✓	✓
Lionel	Male	84	Spouse	Stroke. Cannot walk unaided. 4 care visits a day for personal needs	Day Centre	✓	✓	✓
Violet	Female	90	Alone	Registered blind. Limited mobility. 3 care visits a day for general and personal needs	Day Centre	✓	X	X

### 2.2 Setting

Interviews took place in participants' homes and between care visits, to ensure privacy and avoid intrusion. Since diary entries could refer to formal carers' actions, SH suggested that diaries be kept in a private place. At any sign of fatigue or distress, participants were offered comfort breaks or asked if they wished to continue at a later date.

### 2.3 Limitations of the study

The sample was not diverse in terms of ethnicity (although it was representative of the local population) or gender. Reflecting other researchers' difficulties in recruiting men to similar studies (Equality & Human Rights Commission, 2011; Gethin‐Jones, 2012), all but three of the seventeen participants were women. It also proved difficult to reach those who were unable to leave their homes due to frailty and poor physical functioning.

### 2.4 Ethical approval

Ethical approval was granted by the University's Faculty Research Ethics Panel in November 2013, and data collection occurred during 2014. Following initial contact, SH visited participants to introduce the study and ensure consent was fully informed. Written consent was obtained in all instances.

### 2.5 Data collection

Data collection was a three‐stage process.

#### 2.5.1 Stage 1—Narrative‐biographical interview

Participants were asked to talk about their lives as they aged and how they managed any transition from independence to being in a relationship of care. A biographical‐narrative approach provides insight into the narrator's social and cultural identity as they construct stories about their life (McAdams, 2001; Pasupathi, Mansour, & Brubaker, 2007).

#### 2.5.2 Stage 2—Diary

Participants were each provided with a notebook and asked to record daily thoughts and reflections about their formal care over a period of 14 days. The diary is another narrative tool, yielding rich qualitative data that tells the participant's own story and can be used to ‘record an ever‐changing present’ (Plummer, 2001; p.48).

#### 2.5.3 Stage 3—Follow‐up interview

The follow‐up interview enabled emergent issues to be explored, focusing on how participants felt about the incidents they recorded in the diary and any interaction between care practices and sense of self.

While thirty‐four items of data were obtained, only eight of the seventeen participants completed all three stages of data collection (see Table 1). The range of methods enabled participants to express their thoughts about their care in different ways. Where diaries and follow‐up interviews *were* available, they provided valuable additional insights to the primary vehicle of the biographical‐narrative interview. Nevertheless, each method was a valuable resource in its own right. Completing the diary at home gave participants the opportunity to contextualise entries, writing about events where and when they happened, which improved recall and helped capture events that could be seen as trivial and thus forgotten over time (Verbrugge, 1980). Some used the diary to vent frustrations or process thoughts about their care.

### 2.6 Data analysis

All interviews were audio‐recorded, transcribed and anonymised. Fictitious names were chosen, to give a sense of the persons in the experiences (Given, 2015). Using NVivo, transcripts were reviewed and re‐reviewed, codes were identified and significant themes generated. Diaries were typed up and coded similarly. SH used both the biographical‐narrative and diary themes to prepare a list of common probes and questions for the follow‐up interviews, alongside some individual‐specific items. For example, someone who frequently recorded frustration at carers' lateness in his diary was subsequently asked to expand on this and how it had made him feel as a person. Another, who talked about experiences of lifelong disability requiring care, was later asked how more recent care compared. Follow‐up interviews were then transcribed and analysed in turn. This provided a complete single data set for each participant**.** Coding and themes were reviewed within the research team, in order to support the interpretation of the data and increase credibility (Lincoln & Guba, 1985).

### 2.7 Findings

Three major themes were identified. First, *self, identity and getting older* addressed how older people receiving care at home saw themselves within this context. Second, *relationships with others* reflected the social context of care. Third, *autonomy* emphasised the importance of maintaining control over personal environments.

#### 2.7.1 Theme 1: Self, identity and getting older

Participants were eager to speak about their lives, how they saw themselves as they aged and how they felt about receiving care. Earlier life experiences were seen as key, reflecting a common emphasis on continuity of self. Personality traits, such as integrity and honesty, were highlighted as enduring and influencing current experiences of care. Lionel (84) had been unable to walk unaided since suffering a stroke. His wife (82) was unable to care for him, and so carers visited four times a day to help with washing, dressing, toileting, meal preparation and medication prompts. Throughout his working life, he had held a senior role, and he took great pride in his professionalism and integrity. When carers failed to uphold these values, he felt frustrated and unhappy:

*“See I spent a lot of my working life in sales and if I couldn't fulfil something I said I'd do I'd ring the person up and not leave them to find out, which to me is courtesy. Hmm, I'm not just a number on a sheet, I'm a person”* [Lionel, Follow‐up Interview].

Similarly, Maurice (74) had been severely disabled since a teenager and needed considerable help. He had always been determined to live a full life, presenting himself as smart, able and positive, yet his extreme physical limitations meant that he now needed carers four times a day. However, he was adamant that they should respect his high standards of personal presentation:

*‘At one time I wouldn't ever go out without a tie on …. I don't like to give an appearance where I'm all dishevelled… you don't have to go around in stained clothes'* [Maurice: Follow‐up Interview].

Others highlighted characteristics of endurance and determination, which now helped them in their cared‐for role. For example, Clare (82) who suffered a stroke and multiple sclerosis had experienced extreme childhood poverty, which she believed had equipped her with a will to triumph over adversity, helping her to negotiate multiple sclerosis later in life.

However, this enduring sense of self could be jeopardised by changes brought about by ageing and the need for care. For example, participants spoke about growing vulnerability and fears of intruders, falling, loss of financial control or cognitive decline. Susan (72) had a spinal disc problem and chronic obstructive pulmonary disease; she used an oxygen mask and had a permanent catheter. She noted in her diary that she was taken to the toilet by a carer and left there without any indication of when they would return. Because she was afraid of falling, she had to wait:

*‘I couldn't get up, I was just dumped there, I thought I might be there hours'* [Susan, Follow‐up Interview].

Some felt treated them in a demeaning manner. Being seen as old and inadequate resulted in depersonalised care, as reflected in Lionel's dislike of day centre provision:

*‘It's full of old people and I want a challenge and I don't want to sit in a circle and sing songs'* [Lionel, Follow‐up Interview].

As can be seen, the way in which care is delivered can have a direct impact on personal identity.

Another key element in this theme was the strong link between home and identity. The home assumed increasing significance as horizons shrank due to limited mobility. This was challenged by the presence of formal carers and intrusive care equipment, which reconstructed the home as a site of caregiving, clouding the boundaries between the private and social selves. For example, Maurice's carer threatened his control:

*“…She was coming in and invading our space… she was telling me what I could do and I knew exactly what I was up to and sometimes I've had to say to people ‘I also live here!' “* [Maurice: Follow‐up Interview].

Susan's home, where she had lived with her husband for many years, was very important to her. She found carers intrusive:

*‘They're invading my privacy… they're spoiling it… they come in On my privacy and I don't like that'* [Susan: Biographical‐Narrative].

### 2.8 Theme 2: Relationships with others

While a few saw formal carers as an unwelcome intrusion, many cherished and depended on their visits. Pauline (85), who had limited mobility due to osteoporosis, lived alone and needed daily help. When she fell ill but her carer could not attend, Pauline was frightened; she struggled to feed herself and keep hydrated. Davina (71), a widow who had suffered a stroke, had limited mobility and was dependent on formal care three times a day, found the isolation hard to bear:

*‘I just say I hate my life…. the loneliness…I wave at people and they wave back*’’ [Davina, Biographical‐Narrative].

Clare found the periods in between day centre visits hard:

*‘I've got saturday sunday, monday, on my own… well there's nothing wrong with the care, it's just sitting here alone for so long’* [Clare, Follow‐up Interview].

The caring relationship was as much social as practical. Consistency was vital and emphasised by eleven participants. Repeated visits allowed trusting relationships to flourish, promoting a greater sense of control. Yet consistency of carer was not always possible. Some never knew who would arrive to deliver what could be very personal care.

Susan spoke of her long‐term formal carers with great warmth. In contrast, new carers threatened her dignity. Susan gave examples of not wanting to have a shower with a new carer, and of the way that carers would address her husband rather than her:

*‘Well people come In and look over my head They don't bother with me, I'm not there… they make me feel I shouldn't be here…just not be on this earth’* [Susan, Follow‐up Interview].

Regular carers became familiar with individual routines, which made the process of care feel reliable. In contrast, new carers could cause pain and unhappiness. For example, Maurice's new carers rubbed his legs in the wrong direction causing him discomfort. Regular carers also completed the tasks quicker and had time to talk:

*‘She [formal carer] is here the same time as everyone else but gets everything done, always perfect… and she always has time for a good chat’* [Susan, Diary].

If regular carers could not attend, it was important to participants that they knew beforehand and had been previously introduced to the new carer. Susan explained:

*‘I wish they would tell me who they are…. makes me annoyed and angry because I don't know who I'm talking to. It could be a man or woman off the street. It makes me feel like a baby again’* [Susan, Follow‐up Interview].

### 2.9 Theme 3 Autonomy

Maintaining control over personal environments and lives was a powerful theme. Autonomy was affected by practical aspects of care. Timing was crucial, and unpredictable visits could affect plans for the rest of the day. Clare, for example, missed her day centre:

*‘One time I had to stay at home because my carer was too early and I would have to be in the chair for hours. the driver said you're supposed to be sitting in the wheelchair.’* [Clare, Follow‐up Interview].

Late calls could also impact autonomy:

*‘The people that come to take me to the day centre they have their schedule for driving and that and they like to be on time if I'm lucky enough the carers are oN time’* [Eva (92), Biographical‐Narrative].

For some, it was the uncertainty of when the carer would visit that worried them:

*‘Carers do not call if they're going to be late’* [Lionel, Biographical‐Narrative].

Autonomy was diminished by task‐centred and inflexible care. Working rigidly to a tight list of tasks left participants feeling rushed and carers with little time to sit and talk. Susan reported that carers who found themselves running late would rush her care to compensate. In contrast, when carers took the time to consider other needs, including social support, participants felt valued and more confident.

Some spoke positively about carers going beyond prescribed duties. Janet (82) and Margaret (73) said that their carers did extra little things that made them feel safer, and Lionel was happy when one carer went beyond the care plan and took him outside:

‘If I want to go out anywhere I have to have someone to push me in a wheelchair and the carer who comes in the morning took me out once… it was an additional thing’[Lionel, Biographical‐Narrative].

## 3 DISCUSSION

Participants' accounts repeatedly show that the way care is delivered can affect sense of self, as well as influencing feelings of autonomy. Domiciliary care by its very nature does not occur in a neutral space. Yet if it is solely appraised in terms of cost and quality of service delivery, the home becomes no more than a workplace. The older person feels they have lost control, and the home ‘takes on the characteristics of the skeleton of a house rather than the nurturing nature of a home' (Healey‐Ogden, 2014; p.76). This study demonstrates the value of taking a more nuanced view of domiciliary care, by exploring its meaning for its recipients.

There are three significant considerations which emerge from this study. First, care can play an important role in supporting an older person's sense of self. Second, the caring relationship is central, and this can be promoted by regular formal carers. Third, domiciliary care in itself does not produce autonomy; it is how it is delivered that has major implications for a sense of independence and control. Each of these will be considered in turn.

### 3.1 Understanding and supporting the older person's sense of self

Participants emphasised the relevance of their lives to current experiences of care and ageing, reflecting the value of Erikson's theory which sees tensions at different life stages as necessary for personal development. Their values and preferences had evolved through the challenges they had faced. Care that accepts and acknowledges these challenges can support older people as they move through the later stages. Some participants' identities had been shaped through the process of managing disability for many years, and they needed this to be reflected in how care was delivered, respecting their expertise by experience. The care dynamic could also be responsive to individuals' values, likes and dislikes in a way which spoke to their distinctive identity, as opposed to ascribing a generic status as an older service user, which felt demeaning.

It cannot be assumed that because care is delivered in the home, it automatically reflects an ethos of being ‘person‐centred'. Taking a biographical approach can play a crucial role in care (Clarke, 2000; McCormack, 2001; Taylor, 2003) since it is important to develop a clear picture of what an older person values and how they make sense of events. A narrative approach to care and its relationships develops a more holistic and positive understanding between carer and cared‐for.

Another key message was how differently participants experienced ageing. Some who were more physically able expressed feelings of agelessness, in which the younger identity simply continues into old age (Kaufmann, 1994). Others reflected a ‘mask of ageing' (Featherstone & Hepworth, 1989), where outward physicality obscured the inner subjective younger self. Yet some, with a higher level of disability and requiring considerable care, found their energies depleted by the demands of coping with daily living, and they truly felt old. Andrews (1999) maintains that acceptance of the ageing self offers some relief from trying to remain young and busy in order to be seen as having aged successfully.

The study underlines the importance of home as a site of identity and security, not simply of care delivery. For many, it represents safety and comfort, a place for privacy and retreat (Healey‐Ogden, 2014) and a haven of physical and emotional well‐being (Bender, Clune, & Guruge, 2009). Home was where people spent most of their time. The daily routines and habits which come to personify the self were centred here, yet were subject to disruption by care. Participants felt devalued when their homes were treated as workspaces. The home is assumed to be private, yet when a formal carer enters it becomes governed by external rules (Dyck & O'Brien, 2003), and all it represents is compromised. Care equipment has a medicalising impact, and there is a danger that domiciliary services create mini‐institutions in homes; this is more likely if the focus is purely on completing task lists.

### 3.2 The importance of relationship and the regular carer

When an older person receives formal care, they may need to take on a new role of ‘cared‐for', abandoning or renegotiating previously held roles (Kaufmann & Elder, 2003; Leary & Tangney, 2011; McKenna, Broome, & Liddle, 2007). Within Stryker and Statham's perspective (1985), identity is seen as being constructed through role interaction. Resonating with other studies (Doyle, 2012; Equality & Human Rights Commission, 2011), this research underlines the great importance which older people place on their relationships with carers; the way in which the carer and cared‐for roles operated influenced satisfaction with care. However, some found this role adjustment difficult and tensions arose, particularly when relationships could not develop due to carer turnover (Equality & Human Rights Commission, 2011). A clear message of this study is that relational, person‐centred care delivered by consistent, regular formal carers is important. Participants who developed a trusting and supportive relationship with their carers experienced security, familiarity and friendship. This was particularly important for those who lived alone. However, the relationship also mattered to those participants with more support, as it provided a special friendship at the same time as giving respite to informal carers.

### 3.3 Understanding independence, dependence and autonomy in care

Care has a strong inter‐relationship with autonomy. In this study, it became clear that people receiving domiciliary care were often deprived of decisional autonomy concerning how it was delivered, echoing Boyle's (2004) findings. Yet there was also evidence of scope for decisional autonomy to be enhanced, such as when regular formal carers were familiar with the range of needs of an older person. A major factor was the timing of care visits, which affected participation in social activities, echoing Patmore's (2004) findings. These activities are crucial for people who can be isolated, but choice is limited by timing and availability, with attendance at day centres most affected (Pino et al., 2014). As with other research (Morville & Fjordside, 2016; Palmer et al., 2015; Sykes & Groom, 2011), this study demonstrates that older people are typically expected to manage their lives around the carer's schedule. Uncertain or unreliable visits can cause feelings of frustration, abandonment, fear and anger (Breiholtz, Snellman, & Fagerberg, 2013). Some participants in this study were disappointed and even scared by the lack of communication about call times, fearing that visits might not happen at all, and they would be left without food, medication or clean pads.

Care organisations struggle to maintain the continuity so needed by older people, due to high levels of staff turnover and the frequent juggling of rotas to cover absences (Francis & Netten, 2004). Yet the study also supports the argument that relationships are further impeded by care that rigidly sticks to task lists (Gethin‐Jones, 2012) allowing little room for negotiation.

## 4 CONCLUSION

This study has explored the interaction between older people and formal carers within the home. It has demonstrated the importance of considering domiciliary care in terms of older people's identity, individuality and well‐being, as much as cost‐effectiveness or quality. Appreciating the experiences of older people receiving care at home entails understanding that domiciliary care interacts with the sense of self. There is both continuity and change in the self, as it results from life experience yet is also mediated through role negotiation. This has significant implications for the care dynamic. This study therefore has important messages for practice:

- Care delivery can be enhanced through an understanding of the self, the different experiences of ageing and the value placed on the home space. The biographical approach supports knowledge of the older person through awareness of personal histories, preferences and values. Care plans should go beyond task lists to reflect the older person's unique needs and priorities.
- Care organisations should prioritise allocating regular carers to older people to foster understanding and rapport.
- Carers should acknowledge and understand the difference between executional autonomy and decisional autonomy. Carers restrict the older person's autonomy by making choices on their behalf, yet maintaining decisional autonomy is fundamental to feelings of well‐being (Boyle, 2005). Supporting older people's choices and decisions should be an integral aspect of care.

However, these recommendations present challenges for care organisations. Glendinning (2012; p. 294) suggests that the market is ‘fragmented, unstable and diverse' and the turnover of providers is increasing, while the UKHCA (2016) expresses growing concerns about instability. A rising number of providers are surrendering undeliverable contracts back to local authorities, with fears about the sustainability of their business (UKHCA, 2016). Hence, further research should focus on how organisations operating in this context can best increase awareness of ways in which their procedures and practices directly affect older people receiving care.

In spite of the challenging landscape of home‐care services, care delivery can be improved. When care supports self, identity and autonomy, and when it values the relationship between carer and cared‐for, there is scope to bring about positive change. The implications for resourcing go beyond the scope of this paper. However, what is evident is that the way in which care is delivered has a strong bearing on how well it is received.

## References

[hsc12921-bib-0001] Andrews, M. (1999). The seductiveness of agelessness. Ageing and Society, 19(03), 301–318. 10.1017/S0144686X99007369

[hsc12921-bib-0002] Aronson, J. (2002). Elderly people's accounts of home care rationing: Missing voices in long term care policy debates. Ageing and Society, 22, 399–418. 10.1017/S0144686X02008759 15264341

[hsc12921-bib-0003] Bender, A. , Clune, L. , & Guruge, S. (2009). Considering place in community health nursing. Canadian Journal of Nursing Research, 41, (1), 128–143. Retrieved from https://www.researchgate.net/publication/26255885_Considering_place_in_community_health_nursing/download [Accessed 10 November 2018]. 19485049

[hsc12921-bib-0004] Boyle, G. (2004). Facilitating choice and control for older people in long‐term care. Health and Social Care in the Community, 12(3), 212–220. 10.1111/j.1365-2524.2004.00490.x 19777711

[hsc12921-bib-0005] Boyle, G. (2005). The role of autonomy in explaining mental ill‐health and depression among older people in long‐term care settings. Ageing and Society, 25(5), 731–748. 10.1017/S0144686X05003703

[hsc12921-bib-0006] Breitholtz, A. , Snellman, I. , & Fagerberg, I. (2013). Older people’s dependence on caregivers’ help in their own homes and their lived experiences of their opportunity to make independent decisions. International Journal of Older People Nursing, 8(2), 139–148. 10.1111/j.1748-3743.2012.00338.x 22823527

[hsc12921-bib-0007] Care Act . (2014). (c.23). HSMO: London.

[hsc12921-bib-0008] Clarke, A. (2000). Using biography to enhance the nursing care of older people. British Journal of Nursing, 9, 429–433. 10.12968/bjon.2000.9.7.6323 11111438

[hsc12921-bib-0009] Collopy, B. J. (1988). Autonomy in long term care: Some crucial distinctions. The Gerontologist, 28, 10–17. 10.1093/geront/28.Suppl.10 3139498

[hsc12921-bib-0010] Doyle, S. (2012). Being‐in‐the‐world‐of‐care: The lived experiences of older people receiving community aged care packages in Queensland. Health Care for Women International, 33(10), 905–921. 10.1080/07399332.2012.701256 22946593

[hsc12921-bib-0011] Dyck, I. , & O'Brien, P. (2003). Thinking about environment: Incorporating geographies of disability into rehabilitation science. The Canadian Geographer/Le Géographe Canadien, 47(4), 400–413. 10.1111/j.0008-3658.2003.00032.x

[hsc12921-bib-0012] Equality and Human Rights Commission . (2011). Close to home: An inquiry into older people and human rights in home care. [online]. Retrieved from http://www.equalityhumanrights.com/legal-and-policy/inquiries-and-assessments/inquiry-into-home-care-of-older-people [Accessed 10 November 2018].

[hsc12921-bib-0013] Erikson, E. H. (1982). The life cycle completed. New York: W.W Norton and Company. ISBN: 039301622 6.

[hsc12921-bib-0014] Featherstone, M. , & Hepworth, M. (1989). Ageing and old age: Reflections on the postmodern life course In: Bytheway B. (Ed.), Becoming and being old sociological approaches to later life. London: Sage Publications Ltd. ISBN: 9780803981713.

[hsc12921-bib-0015] Francis, J. , & Netten, A. (2004). Raising the quality of home care: A study of service users’ views. Social Policy and Administration, 38(3), 290–305. 10.1111/j.1467-9515.2004.00391.x

[hsc12921-bib-0016] Gethin‐Jones, S. (2012). Does Outcome‐focused Intervention for Frail Older People Provide Better Quality Care than Current 'Time and Task' Models? PhD, Cardiff University.Retrieved from http://orca.cf.ac.uk/45266/ [Accessed 10 November 2018].

[hsc12921-bib-0017] Given, L. M. (2015). 100 questions (and answers) about qualitative research. United States: SAGE Publications. ISBN: 9781483345642

[hsc12921-bib-0018] Glendinning, C. (2012). Home care in England: Markets in the context of under‐funding. Health & Social Care in the Community, 20(3), 292–299. 10.1111/j.1365-2524.2012.01059.x 22360615

[hsc12921-bib-0019] Glendinning, C. , Clarke, S. , Hare, P. , & Maddison, J. (2008). Progress and problems in developing outcome‐focused social care services for older people in England. Health & Social Care in the Community, 16(1), 54–63. 10.1111/j.1365-2524.2007.00724.x 18181815

[hsc12921-bib-0020] Healey‐Ogden, M. J. (2014). Being “at Home” in the Context of Home Care. Home Health Care Management & Practice, 26(2), 72–79. 10.1177/1084822313508646

[hsc12921-bib-0021] Hvalvik, S. , & Reierson, I. Å. (2011). Transition from self‐supported to supported living: Older people's experiences. International Journal of Qualitative Studies on Health and well‐being, 6(4), 10.3402/qhw.v6i4.7914 PMC3228403 22135699

[hsc12921-bib-0022] Janssen, B. M. , Van Regenmortel, T. , & Abma, T. A. (2011). Identifying sources of strength: Resilience from the perspective of older people receiving long‐term community care. European Journal of Ageing, 8(3), 145–156. 10.1007/s10433-011-0190-8 21949496 PMC3156942

[hsc12921-bib-0023] Karlsson, S. , Edberg, A. , Jakobsson, U. , & Hallberg, I. R. (2013). Care satisfaction among older people receiving public care and service at home or in special accommodation. Journal of Clinical Nursing, 22(3–4), 318–330. 10.1111/jocn.12115 23301576

[hsc12921-bib-0024] Kaufman, G. , & Elder, G. H. Jr . (2003). Grandparenting and age identity. Journal of Aging Studies, 17(3), 269–282. 10.1016/S0890-4065(03)00030-6

[hsc12921-bib-0025] Kaufman, S. R. (1994). The ageless self: Sources of meaning in late life. Madison, WI: University of Wisconsin Press. ISBN: 9780299108649.

[hsc12921-bib-0026] Leary, M. R. , & Tangney, J. P. (Eds.) (2011). Handbook of self and identity. New York, NY: Guilford Press. ISBN: 9781462503056

[hsc12921-bib-0027] Lincoln, Y. S. , Guba, E. G. , & Pilotta, J. J. (1985). Naturalistic inquiry. Beverly Hills, CA: Sage. ISBN 9780803924314.

[hsc12921-bib-0028] Local Government Association’s Well‐being Board . (2018). £8 billion funding black hole by 2025 will swallow up popular council services. [online]. Retrieved from https://www.local.gov.uk/about/news/funding-black-hole. [Accessed 10 November2018].

[hsc12921-bib-0029] McAdams, D. P. (2001). The psychology of life stories. Review of General Psychology, 5(2), 100 10.1037/1089-2680.5.2.100

[hsc12921-bib-0030] McCormack, B. (2001). Negotiating Partnerships with Older People: A Person‐Centred Approach.Ashgate, Aldershot 10.4324/9781315195346

[hsc12921-bib-0031] McKenna, K. , Broome, K. , & Liddle, J. (2007). What older people do: Time use and exploring the link between role participation and life satisfaction in people aged 65 years and over. Australian Occupational Therapy Journal, 54(4), 273–328. 10.1111/j.1440-1630.2007.00642.x

[hsc12921-bib-0033] Morville, A. , & Fjordside, S. (2016). Factors influencing older people's experiences of participation in autonomous decisions concerning their daily care in their own homes: A review of the literature. International Journal of Older People Nursing, 11(4), 284–297. 10.1111/opn.12116 27019374

[hsc12921-bib-0034] Office for National Statistics . (2017). Overview of the UK population: July 2017. [online]. Retrieved from https://www.ons.gov.uk/releases/overviewoftheukpopulationjuly2017 [Accessed 10 November 2018].

[hsc12921-bib-0035] Palmer, D. , Williams, L. , Hatzidimitriadou, E. , Hossain, R. , Ball, C. , Rigby, N. , … Hinds‐Murray, A. (2015). Care for me at home. A qualitative exploration of experiences of people receiving domiciliary (home) care in the london borough of bexley. [online] Retrieved from http://www.healthwatchbexley.co.uk/sites/default/files/care_for_me_at_home_full_report.pdf[Accessed 24 October 2018].

[hsc12921-bib-0036] Pasupathi, M. , Mansour, E. , & Brubaker, J. R. (2007). Developing a life story: Constructing relations between self and experience in autobiographical narratives. Human Development, 50(2–3), 85–110. 10.1159/000100939

[hsc12921-bib-0037] Patmore, C. (2004). Quality in home care for older people: Factors to pay heed to. Quality in Ageing Policy: Practice and Research, 5(1), 32–40. 10.1108/14717794200400005

[hsc12921-bib-0038] Patmore, C. , & McNulty, A. (2005). Making Home Care for Older People more Flexible and Person‐Centred. [online] Available at https://www.bl.uk/collection-items/making-home-care-for-older-people-more-flexible-and-personcentred-factors-which-promote-this[Accessed 15 October 2018].

[hsc12921-bib-0039] Pino, L. , González‐Vélez, A. E. , Prieto‐Flores, M. E. , Ayala, A. , Fernandez‐Mayoralas, G. , Rojo‐Perez, F. , … Forjaz, M. J. (2014). Self‐perceived health and quality of life by activity status in community‐dwelling older adults. Geriatrics & Gerontology International, 14(2), 464–473. 10.1111/ggi.12119 23890252

[hsc12921-bib-0040] Plummer, K. (2001). Documents of life 2: An invitation to a critical humanism, 2. London: Sage Publications 10.1177/1525822X0101300306

[hsc12921-bib-0041] Qureshi, H. , & Henwood, M. (2000). Older people's definitions of quality services. [online] Available at http://citeseerx.ist.psu.edu/viewdoc/download?doi=10.1.1.492.2138%26rep=rep1%26type=pdf[Accessed 15 October 2018].

[hsc12921-bib-0042] Sixsmith, A. , & Sixsmith, J. (2008). Ageing in place in the United Kingdom. Ageing International, 32(3), 219–235. 10.1007/s12126-008-9019-y

[hsc12921-bib-0043] Stryker, S. , & Statham, A. (1985). Symbolic interaction and role theory In: Lindzey G., & Aronson E. (Eds.), Handbook of Social Psychology (pp. 311–378). New York: Random House 10.14966/jssp.KJ00003724954

[hsc12921-bib-0044] Sykes, W. , & Groom, C. (2011). Older people’s experience of home care in England. London: Equality and Human Rights Commission [online]. Retrieved from https://www.equalityhumanrights.com/sites/default/files/research-report-79-older-peoples-experiences-of-home-care-in-england.pdf[Accessed 09 October 2018].

[hsc12921-bib-0045] Taylor, C. (2003). Narrating practice: Reflective accounts and the textual construction of reality. Journal of Advanced Nursing, 42(3), 244–251. 10.1046/j.1365-2648.2003.02613.x 12680968

[hsc12921-bib-0046] Trust, N. . (2017). The autumn budget: Joint statement on health and social care. [online]. Retrieved from https://www.nuffieldtrust.org.uk/research/autumn-budget-2017[Accessed 10 November 2018].

[hsc12921-bib-0047] United Kingdom Home Care Association . (2016). An Overview of the Domiciliary Care Market in the United Kingdom. [online]. Retrieved from https://www.ukhca.co.uk/pdfs/DomiciliaryCareMarketOverview2015.pdf[Accessed 11 October 2018].

[hsc12921-bib-0048] Verbrugge, L. M. (1980). Health Diaries. Care, 1, 73–95. 10.1097/00005650-198001000-00006 6986517

